# Practice nursing in Australia: A review of education and career pathways

**DOI:** 10.1186/1472-6955-8-5

**Published:** 2009-05-27

**Authors:** Rhian M Parker, Helen M Keleher, Karen Francis, Omar Abdulwadud

**Affiliations:** 1 Australian Primary Health Care Research Institute, Australian National University, Acton, ACT, 0200, Australia; 2 Department of Health Science, Monash University Peninsula Campus, Frankston, Victoria, 3195, Australia; 3 School of Nursing and Midwifery, Monash University, Gippsland Campus Northways Road, Churchill, Victoria 3842, Australia

## Abstract

**Background:**

Nurses in Australia are often not educated in their pre registration years to meet the needs of primary care. Careers in primary care may not be as attractive to nursing graduates as high-tech settings such as intensive or acute care. Yet, it is in primary care that increasingly complex health problems are managed. The Australian government has invested in incentives for general practices to employ practice nurses. However, no policy framework has been developed for practice nursing to support career development and post-registration education and training programs are developed in an ad hoc manner and are not underpinned by core professional competencies. This paper reports on a systematic review undertaken to establish the available evidence on education models and career pathways with a view to enhancing recruitment and retention of practice nurses in primary care in Australia.

**Methods:**

Search terms describing education models, career pathways and policy associated with primary care (practice) nursing were established. These search terms were used to search electronic databases. The search strategy identified 1394 citations of which 408 addressed one or more of the key search terms on policy, education and career pathways. Grey literature from the UK and New Zealand internet sites were sourced and examined. The UK and New Zealand Internet sites were selected because they have well established and advanced developments in education and career pathways for practice nurses.

Two reviewers examined titles, abstracts and studies, based on inclusion and exclusion criteria. Disagreement between the reviewers was resolved by consensus or by a third reviewer.

**Results:**

Significant advances have been made in New Zealand and the UK towards strengthening frameworks for primary care nursing education and career pathways. However, in Australia there is no policy at national level prepare nurses to work in primary care sector and no framework for education or career pathways for nurses working in that sector.

**Conclusion:**

There is a need for national training standards and a process of accreditation for practice nursing in Australia to support the development of a responsive and sustainable nursing workforce in primary care and to provide quality education and career pathways.

## Background

In Australia, courses prepare nurses largely for employment in the acute care sector (medical/surgical units). Preparation and understanding of nursing practice in the broader areas, such as health promotion and preventive care, is limited within most undergraduate curricula in Australian universities. Thus, careers in primary care settings, such as general practice, may not be as attractive as settings such as intensive care or acute care. Yet, it is in primary care that increasingly complex health problems are managed.

Nurses in general practice are now being recognised as a resource for increasing access to primary care and a vehicle for alternative models of health care delivery that can relieve the pressure on general practice. Competency standards[1] for these nurses were developed in 2005 by the Australian Nursing Federation and these are reflective of the Australian Nursing and Midwifery Council competency standards for registered nurses. However, we did not identify any literature that reviewed or evaluated these standards.

Policy developments driving changes in the delivery of primary care are accompanied by funding mechanisms designed to support expansion of practice nursing, while general practice reforms aim to increase their capacity to manage complex health care conditions. Practice nurses (PNs) are a core component of those reforms. As the scale of unmet need for primary care emerges, the contributions that appropriately educated and skilled nurses can make as case managers, coordinators and providers of care, and in preventative health care programs, are being recognised.

The Australian Government defines a practice nurse as a registered or enrolled nurse employed by, or whose services are retained by, a general practice in a general practice may be either accredited or non-accredited [2]. The PN must be appropriately qualified for the services provided and must comply with any relevant legislative or regulatory requirements.

The *2007 National Practice Nurse Workforce Survey *[3]demonstrates that nurses are increasingly regarded as core members of general practice teams. In 2007 there were 7824 nurses employed in Australian general practices[2]However, the attraction and retention of a new generation of nurses to primary care is dependent on the quality of their educational preparation as well as the attractiveness and availability of career pathways. No policy framework has been developed for practice nursing to support career development, while post-registration education and training programs are developed in a somewhat ad hoc manner and are not underpinned by core professional competencies. Education and career pathways are not designed for primary care settings. To attract and retain nurses to work outside traditional acute care settings, comparable conditions are critical. An evidence-informed understanding of options available to increase the attractiveness of primary care nursing can assist policy makers to plan effectively for nursing education in Australia and for primary care and preventative health policy.

This paper reports on a systematic review that was undertaken to establish the available evidence on education models and career pathways with a view to enhancing recruitment and retention of practice nurses in primary care settings. The aim was to examine measures in place to ensure Australia has a capable, efficient and effective primary care nursing workforce to address current and emerging health needs.

The data reported in this paper was part of a wider study that drew on 'linkage and exchange'[4] philosophies to inform the research, to increase the policy relevance of the research questions and to strengthen the evidence base that informs policy. The study also examined health outcomes from primary care nursing and these findings are reported elsewhere [5]

## Methods

### Research question

The research question for the component of the review reported here was: What education models and policy frameworks support career pathways and enhance recruitment and retention of nurses to primary (including practice nursing) and community care nursing? These broad terms were identified so as not to exclude literature from jurisdictions that do not describe nurses working in general practice or similar settings as practice nurses.

### Search strategy

This review followed the methods described by the Cochrane Collaboration. We developed a protocol for the review, including the review question, search methods, inclusion and exclusion criteria, and the approach to assessment and data synthesis. The core terms used in the search included 'primary nursing care', 'community health nursing', 'community health nurse', 'family nurse practitioners', 'practice nurse', 'district nursing', 'office nursing', 'nursing education', 'education programs', 'educational models', 'government policy making', 'government regulation', 'health policy', 'health care policy', 'policy making', and 'health care reform'. The search terms were combined using the Boolean operators 'and', 'or' and 'not'. The search strategy was modified and adapted for each database. In February 2007, searches were conducted to identify English articles published between 1975 and 2007 in a number of electronic databases that included Embase, Medline, CINAHL, PsycINFO, ISI Web of Science, Australian Medical Index, EBM Reviews, via OVID and PubMed. We also searched for the grey literature from the UK and New Zealand internet sites and examined the references of retrieved studies. The UK and New Zealand Internet sites were selected because they have well established and advanced developments in education and career pathways for practice nurses.

Studies meeting the following criteria were included: (a) published studies, reviews or discussion papers addressing the review topics; (b) grey literature; (c) articles published in English language; (d) studies originating from Australia, New Zealand, the United Kingdom, Canada, USA, Europe, Japan, Brazil, South Pacific Nations, Thailand, Malaysia, and Myanmar. These countries were included so as to glean the widest possible literature from different parts of the world.

Two reviewers examined titles, abstracts and studies, based on inclusion and exclusion criteria. Disagreement between the reviewers was resolved by consensus or by a third reviewer.

## Results and Discussion

The search strategy identified 1394 citations of which 408 addressed one or more of the key search terms on policy, education and career pathways. However, most of these failed to meet at least one of the inclusion criteria, and were excluded. Moreover no high-level studies were identified in relation to the review topics and no studies were reported. There was a considerable literature of opinion and discussion papers about primary care nursing and this grey literature provides the evidence in this area. A total of 24 papers and other sources were reviewed. A narrative synthesis [6] approach was used to synthesise the outcomes of papers and reports identified. The following section reports on the evidence gleaned from this synthesis, detailing findings under three main headings: scope of nursing practice, education and training and career pathways.

### Scope of nursing practice in primary care settings

The PN role is essentially envisaged as a complement to the general practitioner, in part to extend the activities of general practice (nurse-led services) and in part to substitute for the general practitioner (GP) (nurses as supplements). There is considerable variation in the actual tasks undertaken by PNs, their level of responsibility and their models of practice, as well as the extent to which the PN is a true 'partner in care' or more of an assistant to the GP. Sibbald et al [7,10] suggest that ...'extending nursing roles in primary care is a plausible strategy for improving service capacity without compromising quality of care or health outcomes for patients'.

PNs undertake a wide range of tasks in different dimensions of responsibility that are constant, irrespective of geographic location. [8] (Table 1)

**Table 1:** Practice nurse responsibilities

*Clinical care *– responsibility for clinical based procedures and activities Specific clinical activities as part of the care team such as assessment of risk factors, lifestyle screening, brief interventions, counselling and education, vaccination, wound care, cervical screening
*Patient follow-up and recall *– both arranging and undertaking follow-up tasks, especially in context of chronic disease management and prevention

*Care planning *– setting up care planning meetings, completing care plans

*Treatment rooms tasks*

*Clinical organization *– activities that require management, coordination and higher level administration of clinical activities, particularly a systems approach

*Practice administration *– activities that provide administrative support to the general practice as a business enterprise

*Integration *– development of effective communication channels within the practice and between the practice and outside organizations and individuals.

In addition, studies identified that PN knowledge should include fire safety, life support, infection control, child protection, and health and safety, with OHS requirements to ensure practice nurses are cognizant of their need to be safe and current in their practice. [9-11]

An RACGP/RCNA evaluation predicted that in the future, PNs will undertake a greater integration role with more time spent in clinical care and clinical organisation, and less time on practice administration [8] PNs themselves believe curriculum content should have breadth to cover a range of skills set out in Table 2[11,12].

**Table 2:** Skills identified for nursing in general practice

*Communication skills *– written, verbal, patient advocacy and conflict resolution, dealing with difficult clients
*Legal and ethical issues*-including confidentiality and national privacy principles

*Infection control *– wound care and management

first aid and CPR

*Chronic disease management *– physical assessment, palliative and end of life care

*Cold chain monitoring*

*Sterilization*

*Triage*

*Prevention and health education *– counselling, health education and promotion, family planning, child health, screening, immunization, mental health, drug (including tobacco) and alcohol screening and brief interventions

*Management skills *– case management, practice accreditation, information technology, recall/reminder register.

### Education and Training

Most PNs are registered nurses (Div 1) with many having a post-registration qualification – predominantly midwifery, and maternal and child health nursing [12]; [11]. However, in one Australian study, over one third of PNs had no post-registration qualifications [8,12]. There is no mandatory training for PNs and a fairly poor infrastructure to provide mandatory training if it were required [9,13].

In Australia, there is little or no prerequisite educational preparation required for nurses who wish to practice as a PN [14]. There are some educational providers who offer post-graduate courses targeting practice nurses but these programs are not mandatory for employment and the uptake of these programs is low [14]. PNs access to informal education is predominantly delivered by general practices themselves or the local Division of General Practice, and is focused on the National Health Priorities which is more appropriate for registered rather than enrolled nurses [11].

In the 2001/2002 Federal Budget the Australian Government allocated a total of $104.3 million over four years for the Nursing in General Practice Programme (NiGP). Of this $86.6 million was allocated to Practice Nurse Incentive Payments (PIP) in rural and remote areas to support the employment of PNs; $12.5 million for a training and support scheme and $5.2 million to the Remote and Rural Nursing Re-entry and Up-skilling Scholarship Scheme [2]. In the 2005–06 Federal Budget a further $129.9 million was provided of which $112.4 million was for an extended PIP scheme and $15.6 million was for training and support. In total over $28 million has been allocated over eight years to support PN education and training [2]

However, no studies were found that demonstrated impact or outcomes from these expenditures on education and training, While there is support for the development of a training and support strategy for PNs [15,10] to date, no consistent training standards and models of career pathways have been developed.

### Career pathways

There are perceptions that Australian nursing faculties regard some types of nursing practice as more legitimate than others. This is coupled with limited exposure to practice environs beyond the acute care sector in undergraduate curriculum. These perceptions are influential on student career choices [16]. A lack of career development in non-acute and community based and primary care contexts of practice has limited the attractiveness of employment in these settings [14]Career pathways with associated rewards are vital if practice nursing jobs are to be attractive [17,18]. Multi-country experience has demonstrated that career progression, dependent on the demonstration of advanced knowledge and practice specific to the field of clinical expertise, is a strong incentive for nurses to remain in the workforce [19,20].

Nurses choose to work in general practices for many reasons that include part-time employment, flexible working hours, and employment close to home. [10-12,21,22] Although some have argued that Australia's practice nurses have been largely unconcerned with career advancement [11], this milieu is changing as practice nurses' scope of practice expands and this context of practice gains recognition by government and the profession as a legitimate primary care nursing specialism [11,12,23]. There are also significant health workforce shortages in Australia and practice nurses are becoming increasingly important to the delivery of primary care services.

In the United Kingdom (UK) and New Zealand, career trajectories have been developed to progress and support general practice nursing [11] The development of advanced nurse roles in primary care in the UK is argued as 'a plausible strategy for improving service capacity without compromising the quality of care or health outcomes for patients' [7] The development of nurses' scope of practice is extending in primary care with nurse-led clinics, walk-in centres, and health advice by telephone, whilst nurses increasingly substitute for general practitioners in the care and routine management of minor and chronic illness. The career framework developed in the UK is linked to competencies and is outlined in Table 3.

**Table 3:** UK General Practice Nursing Career Framework[27]

**Level**	**Nomenclature**
Level 9	Nurse Partner
Level 8	Advanced Nurse Practitioner
Level 7	Lead General Practice Nurse
Level 6	Senior General Practice Nurse
Level 5	General Practice Nurse
Level 4	Assistant Practitioner
Level 3	Senior Health Care Assistant
Level 2	Health Care Assistant
Level 1	Initial Entry jobs

In New Zealand, a national Primary Health Care Strategy has identified primary health care nurses as crucial to its successful implementation. In 2001, the Strategy saw that a framework for nurses was needed to 'facilitate a national approach to primary health care nursing that would address the capabilities, responsibilities and areas of professional practice, as well as setting educational and career frameworks and exploring suitable employment arrangements' [24]. Since 2001, the New Zealand approach has recognised the potential for enhanced roles for the involvement of practice nurses to align primary health care nursing practice with community need; to develop nursing leadership for new roles and models of practice; nurses involvement in the governance of PHOs; and developing a national career pathway for primary health care nurses, as well as advanced nursing programmes and nurse practitioner programmes [24]

In Australia, there is no career pathway for nurses working in the general practice sector and no incentives to improve skills and enhance their role in the delivery of primary care. Similarly, remuneration is variable [25] and does not seem to be linked to the nurse's skill and clinical expertise. Perhaps this is related to the relative newness of this field, or perceptions that practice nurses have a limited scope of practice which does not encompass complexity and therefore does not require a comprehensive competency, education and career framework[12]. However, there is recognition that a career pathway needs to be developed [11]. Much of the Australian literature equates access to education as being equivalent to providing a career pathway [22,26] but the experience of the United Kingdom is that those career pathways need to be linked to competencies with knowledge and skill development appropriate to articulated career levels. This is consistent with other nursing specialisms such as intensive care nursing or maternal and child health nursing. In order to ensure that we have a well trained and committed nursing workforce in the primary care setting, a nationally coordinated approach needs to be developed. This would focus on the implementing of a career framework for practice nursing based on education levels and competencies and skills to support the professionalisation of practice nursing so as to attract and retain nurses into the sectors, and to support health reforms that aim to increase access to primary care services.

The ageing of the Australian population, the increase in chronic disease and the shift of care from hospitals to the community has increased the demand for primary care services. At the same time, Australia is facing health workforce supply challenges at all levels. The Australian government has adopted a range of strategies for addressing workforce shortages in primary care and one of these strategies is to develop the role of the nurse and expand the clinical tasks nurses carry out in general practice in particular.

This review has found that practice nurse education in Australia is mostly informal and unaccredited and that there are no guidelines for the minimum education requirements for practice nursing relative to competencies [1] and a career structure. Enabling nurses to work effectively in general practice requires that they are properly educated for their role and that there is a competency based career pathway. Effective policies need to be implemented at national level to address these issues and facilitate the development of the role of nurses in primary care in Australia.

Significant advances have been made in New Zealand, the UK and elsewhere towards strengthening frameworks for primary nursing education to support policy shifts towards primary care, and to meet community needs for community based service delivery. The United Kingdom career framework for practice nurses provides an exemplary model tying competencies to education and career pathways. Increased value could be derived from primary care nurses if a systematic career and education framework were in place in Australia. Nurses' sense of job satisfaction and achievement is tied to career development, education, training and professional autonomy. Recruitment and retention are intimately tied to these workforce factors which are neglected relative to other forms of nursing and other types of health professionals.

## Conclusion

There is a need for national training standards for practice nursing and a process of accreditation in Australia to support the development of a responsive and sustainable nursing workforce in primary care. Whilst substantial funding has been made available for the training of practice nurses in specified clinical skills, there are no outcomes frameworks for that education, and it is very limited in its scope. Moreover, current pre-registration education in Australia does not prepare nurses for primary care while postgraduate education is piecemeal, not comprehensive or consistent across the country and also lacks quality assured outcome evaluation. The experience of the UK and NZ is that career frameworks are necessary for recruitment and retention, that is, to attract nurses into primary care and retain the services of nurses who are already working in the sector as well as acknowledging the accumulation of skills and knowledge that comes with experience and education. For efficiency and effectiveness, remuneration is best linked to skill levels and education and the tasks undertaken by nurses at those different skill levels.

## Competing interests

The authors declare that they have no competing interests.

## Authors' contributions

RMP was one of the reviewers on the systematic review team and drafted the manuscript. HMK and KF were reviewers on the systematic review team and commented on the manuscript. OA conducted the searches for the systematic review.

## Pre-publication history

The pre-publication history for this paper can be accessed here:


